# Upregulation of BST-2 by Type I Interferons Reduces the Capacity of Vpu To Protect HIV-1-Infected Cells from NK Cell Responses

**DOI:** 10.1128/mBio.01113-19

**Published:** 2019-06-18

**Authors:** Jérémie Prévost, Suzanne Pickering, Mitchell J. Mumby, Halima Medjahed, Gabrielle Gendron-Lepage, Gloria G. Delgado, Brennan S. Dirk, Jimmy D. Dikeakos, Christina M. Stürzel, Daniel Sauter, Frank Kirchhoff, Frederic Bibollet-Ruche, Beatrice H. Hahn, Mathieu Dubé, Daniel E. Kaufmann, Stuart J. D. Neil, Andrés Finzi, Jonathan Richard

**Affiliations:** a Centre de Recherche du CHUM, Montreal, Quebec, Canada; b Département de Microbiologie, Infectiologie et Immunologie, Université de Montréal, Montreal, Québec, Canada; c Department of Infectious Disease, King’s College London School of Life Sciences and Medicine, Guy’s Hospital, London, United Kingdom; d Department of Microbiology and Immunology, Schulich School of Medicine and Dentistry, The University of Western Ontario, London, Ontario, Canada; e Institute of Molecular Virology, Ulm University Medical Center, Ulm, Germany; f Department of Medicine, Perelman School of Medicine, University of Pennsylvania, Philadelphia, Pennsylvania, USA; g Department of Microbiology, Perelman School of Medicine, University of Pennsylvania, Philadelphia, Pennsylvania, USA; h Department of Medicine, Université de Montréal, Montreal, Quebec, Canada; i Center for HIV/AIDS Vaccine Immunology and Immunogen Discovery, The Scripps Research Institute, La Jolla, California, USA; j Department of Microbiology and Immunology, McGill University, Montreal, Quebec, Canada; Columbia University/HHMI; Icahn School of Medicine at Mount Sinai; University of California, San Diego

**Keywords:** ADCC, DNAM-1, HIV, NK cells, NTB-A, PVR, Vpu, type I IFNs

## Abstract

The HIV-1 accessory protein Vpu enhances viral release by counteracting the restriction factor BST-2. Furthermore, Vpu promotes NK cell evasion by downmodulating cell surface NTB-A and PVR, known ligands of the NK cell receptors NTB-A and DNAM-1, respectively. While it has been established that Vpu’s transmembrane domain (TMD) is required for the interaction and intracellular sequestration of BST-2, NTB-A, and PVR, it remains unclear how Vpu manages to target these proteins simultaneously. In this study, we show that upon upregulation, BST-2 is preferentially downregulated by Vpu over its other TMD substrates. We found that type I interferon (IFN)-mediated BST-2 upregulation greatly impairs the ability of Vpu to downregulate NTB-A and PVR. Our results suggest that occupation of Vpu by BST-2 affects its ability to downregulate other TMD substrates. Accordingly, knockdown of BST-2 increases Vpu’s potency to downmodulate NTB-A and PVR in the presence of type I IFN treatment. Moreover, we show that expression of human BST-2, but not that of the macaque orthologue, decreases Vpu’s capacity to downregulate NTB-A. Importantly, we show that type I IFNs efficiently sensitize HIV-1-infected cells to NTB-A- and DNAM-1-mediated direct and antibody-dependent NK cell responses. Altogether, our results reveal that type I IFNs decrease Vpu’s polyfunctionality, thus reducing its capacity to protect HIV-1-infected cells from NK cell responses.

## Abstract (precis)

The restriction factor BST-2 and the NK cell ligands NTB-A and PVR are among a growing list of membrane proteins found to be downregulated by HIV-1 Vpu. BST-2 antagonism enhances viral release, while NTB-A and PVR downmodulation contributes to NK cell evasion. However, it remains unclear how Vpu can target multiple cellular factors simultaneously. Here we provide evidence that under physiological conditions, BST-2 is preferentially targeted by Vpu over NTB-A and PVR. Specifically, we show that type I IFNs decrease Vpu’s polyfunctionality by upregulating BST-2, thus reducing its capacity to protect HIV-1-infected cells from NK cell responses. This indicates that there is a hierarchy of Vpu substrates upon IFN treatment, revealing that for the virus, targeting BST-2 as part of its resistance to IFN takes precedence over evading NK cell responses. This reveals a potential weakness in HIV-1’s immunoevasion mechanisms that may be exploited therapeutically to harness NK cell responses against HIV-1.

## INTRODUCTION

Robust type I interferon (IFN) responses are among the earliest host immune defenses observed during acute HIV-1 infection (1). Interferons play a central role in early antiviral immune responses and efficiently suppress HIV-1 replication *in vitro*. A part of their antiviral activity depends on the induction of interferon-stimulated genes (ISGs), including restriction factors, such as APOBEC3, TRIM5, SAMHD1, or BST-2 (bone marrow stromal cell antigen 2), that inhibit various steps of the viral replication cycle (2). For instance, BST-2, also known as Tetherin, CD317, or HM1.24, inhibits the release of HIV-1 by trapping newly formed virions at the surface of infected cells (3, 4). BST-2 is a dimeric glycoprotein that consists of an N-terminal cytoplasmic tail, a conventional transmembrane (TM) alpha-helix, a coiled-coil extracellular domain, and a C-terminal glycosylphosphatidylinositol (GPI) anchor (5, 6). This unique topology allows BST-2 dimers to cross-link budding virions at the surface of infected cells by incorporating one of their membrane-associated domains into the viral membrane, preferentially the GPI anchor, while the other membrane-associated domain remains associated with the cell membrane (7).

BST-2 antagonism is a highly conserved function among primate lentiviruses (8). In the case of HIV-1, the small accessory protein Vpu acts as a viral antagonist of BST-2 (3, 4). Vpu-mediated BST-2 downregulation requires a physical interaction between the transmembrane domains (TMDs) of the two proteins (9–11). This interaction is mediated by residues A10, A14, A18, and W22 from the highly conserved alanine face of the TMD of Vpu (9, 10). Although the precise mechanism(s) used by Vpu to antagonize BST-2 is not completely understood, the current consensus points toward a subversion of BST-2 trafficking. Vpu has been shown to block newly synthesized as well as recycled BST-2 from trafficking to the plasma membrane (12, 13). To achieve this, Vpu hijacks the AP-1-dependent membrane trafficking pathway and forces BST-2 into clathrin-rich domains of endosomes and the *trans*-Golgi network (TGN) (14–16). Subsequently, Vpu targets BST-2 to an ESCRT-dependent endosomal degradation pathway through a mechanism that depends on a highly conserved DSGNES motif located in the cytoplasmic tail of Vpu (17–19). Phosphorylation of the serine residues of this motif allows the recruitment of the β-TrCP subunit of the SCF^β-TrCP1/2^ E3 ubiquitin ligase complex, leading to the ubiquitination and degradation of BST-2 in lysosomes (20, 21). Increasing evidence suggests that the recruitment of β-TrCP and the degradation of BST-2 by Vpu are dissociable from its ability to antagonize BST-2 (11, 14, 15, 18, 19, 22), although this is subject to debate (23).

In addition to BST-2, Vpu downmodulates a variety of cellular transmembrane proteins, including the viral receptor CD4 (24, 25) and proteins involved in NK cell-mediated immune responses (26, 27). Vpu contributes to NK cell evasion by downmodulating the NK cell ligands NTB-A and PVR from the surface of HIV-1-infected cells (26, 27). NTB-A (NK, T, and B cell antigen, also known as CD352 and SLAMF6) is a type 1 TM protein that belongs to the signaling lymphocytic activation molecules (SLAM) family of receptors found on the surface of all human NK, T, and B cells (28). One interesting feature of this family of receptors is their preference for homophilic interactions. In NK cells, NTB-A functions as a coactivating receptor, and a homophilic NTB-A–NTB-A interaction triggers NK cell cytotoxicity (29, 30). The downregulation of NTB-A from the surface of HIV-1-infected cells by Vpu was found to prevent NK cell degranulation, thus protecting infected cells from NK cell-mediated lysis (27). Unlike BST-2, Vpu does not enhance NTB-A degradation (27, 31). Instead, Vpu seems to alter the anterograde trafficking of NTB-A, resulting in its sequestration in perinuclear compartments (31). Vpu’s anti-NTB-A activity also appears to require a physical interaction between the TMDs of the two proteins (27).

PVR (polio virus receptor, also known as CD155) is a nectin-like protein that serves as a specific ligand for DNAM-1 (DNAX accessory molecule 1, also known as CD226), an adhesion molecule expressed by multiple cell types, including NK and CD8^+^ T cells. DNAM-1 functions as an activating NK cell receptor, and consequently, its stimulation enhances NK cell-mediated cytotoxicity and cytokine production (32, 33). To reduce the susceptibility of HIV-1-infected cells to NK cell-mediated lysis, Vpu and Nef were shown to downregulate cell surface PVR in an additive manner (26). Similar to the fate of NTB-A in the presence of Vpu, PVR accumulates within perinuclear compartments upon Vpu expression (34). Interestingly, Vpu-mediated downregulation of PVR depends on the same key TM residues, A10, A14, and A18, known to be crucial for BST-2 interaction (34).

While it has been established that Vpu’s TMD is required for the interaction and intracellular sequestration of BST-2, NTB-A, and PVR, it remains unclear how Vpu manages to target these different proteins using the same TMD. In this study, we show that the upregulation of BST-2 by type I IFNs greatly impairs the ability of Vpu to downregulate NTB-A and PVR from the surface of infected primary CD4^+^ T cells. Accumulation of NTB-A and PVR at the surface of HIV-1-infected cells leads to an enhancement in direct and antibody (Ab)-dependent NK cell-mediated killing.

## RESULTS

### Type I IFNs enhance NTB-A and PVR levels at the surface of HIV-1-infected cells.

**FIG 1:**
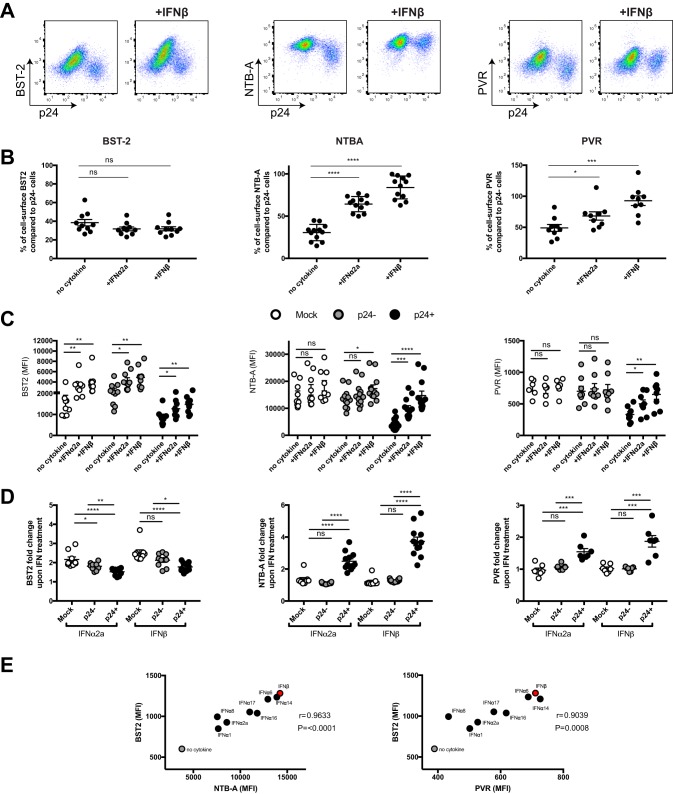
Type I IFNs enhance cell surface NTB-A and PVR on HIV-1-infected cells. Primary CD4^+^ T cells were mock infected or infected with the CH58 transmitted/founder virus (CH58 TF) and either mock treated or treated for 24 h with type I IFNs (IFN-α, IFN-β). At 48 h postinfection, cells were stained with anti-BST-2, anti-NTB-A, or anti-PVR Abs, followed by the appropriate secondary Abs. (A) Dot plot depicting representative stainings with or without IFN-β treatment. (B to D) The graphs shown represent the percentage of median fluorescence intensities (MFI) detected on the p24^+^ population over the MFI detected on the p24^−^ population (B), the MFI obtained on mock-infected cells, uninfected p24^−^ cells, or infected p24^+^ cells (C), or the fold change in MFI upon IFN treatment for at least 6 independent experiments (D). Error bars indicate means ± standard errors of the means (SEM). Statistical significance was tested using an unpaired *t* test or the Mann-Whitney test based on statistical normality (*, *P* < 0.05; **, *P* < 0.01; ***, *P* < 0.001; ****, *P* < 0.0001; ns, nonsignificant). (E) Correlations between the levels of cell surface BST-2 and the levels of NTB-A or PVR on the infected (p24^+^) population upon treatment with different type I IFNs was calculated using a Pearson correlation test.

**FIG 2:**
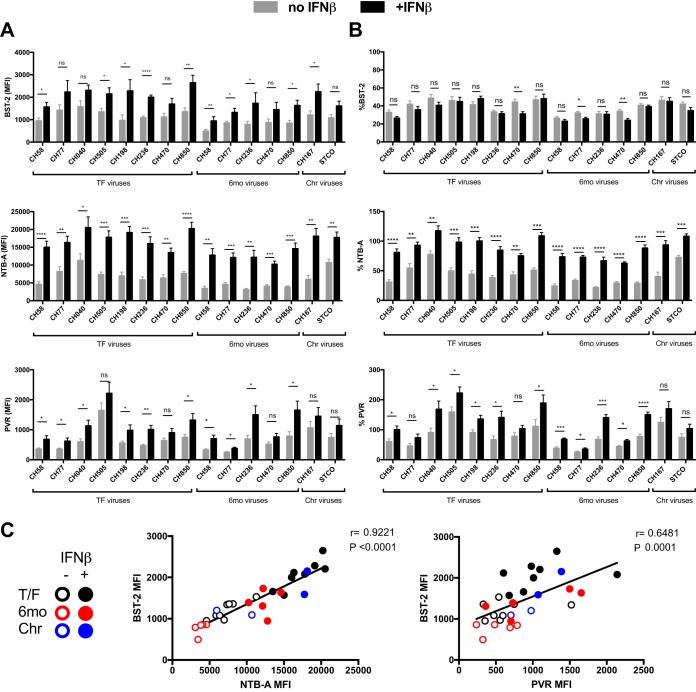
IFN-β enhances cell surface NTB-A and PVR on cells infected with TF, 6-month (6mo), and chronic (Chr) viruses. Primary CD4^+^ T cells were infected with different transmitted/founder (TF) (CH58, CH77, CH40, CH505, CH198, CH236, CH470, CH850), 6-month (CH58, CH77, CH236, CH470, CH850), and chronic viruses (CH167, STCO) and either mock treated or treated for 24 h with IFN-β. At 48 h postinfection, cells were stained with anti-BST-2, anti-NTB-A, or anti-PVR Abs, followed by the appropriate secondary Abs. The graphs shown represent the MFI obtained on infected (p24^+^) cells (A) or the percentage of the MFI on the p24^+^ population over the MFI on the p24^−^ population (B). These graphs represent data obtained from at least 5 independent experiments. Error bars indicate means ± SEM. Statistical significance was tested using an unpaired *t* test or the Mann-Whitney test based on statistical normality (*, *P* < 0.05; **, *P* < 0.01; ***, *P* < 0.001; ****, *P* < 0.0001; ns, nonsignificant). (C) Correlations between the levels of cell surface BST-2 and the levels of NTB-A or PVR on the infected (p24^+^) population of the different viruses tested upon treatment with different type I IFNs were tested using the Spearman rank correlation test.

Since Vpu utilizes the same domain to target BST-2, NTB-A, and PVR, we speculated that the upregulation of one of these substrates could affect/compete with the downregulation of the other substrates. As BST-2 is an ISG, we decided to upregulate its expression using type I IFNs. Activated primary CD4^+^ T cells isolated from healthy HIV-1-negative individuals were mock infected or infected with the transmitted/founder (TF) virus CH58 (CH58 TF). At 24 h postinfection, cells were treated or not with type I IFNs (alpha2a interferon [IFN-α2a] or IFN-β), and the cell surface levels of the three TM proteins were monitored 24 h after IFN treatment by flow cytometry (see Fig. S1 in the supplemental material). In the absence of IFN treatment, the virus was able to downregulate the three TM proteins from the surface of infected (p24-positive [p24^+^]) cells, in contrast to uninfected p24-negative (p24^−^) cells (Fig. 1A and B), in a Vpu-dependent manner (Fig. S2). Strikingly, IFN-α2a or IFN-β treatment significantly abrogated the ability of the virus to downregulate NTB-A and PVR, but not BST-2, from the cell surface (Fig. 1A and B). As previously reported (35), BST-2 levels on all cell populations were significantly enhanced upon IFN-α2a or IFN-β treatment, but this increase was less pronounced on the infected p24^+^ cells (Fig. 1C and D), suggesting that the virus still manages to downregulate this restriction factor upon type I IFN treatment (Fig. 1B). On the other hand, even though NTB-A and PVR levels stayed relatively stable on mock-infected and uninfected bystander cells, the levels for these two ligands at the surface of infected cells were significantly enhanced upon IFN-α2a or IFN-β treatment (Fig. 1C and D). Similar results were obtained with different IFN-α subtypes (IFN-α1, IFN-α6, IFN-α8, IFN-α14, IFN-α16, and IFN-α17), and their ability to upregulate NTB-A and PVR positively correlated with their capacity to enhance BST-2 levels in p24^+^ cells (Fig. 1E). Among all type I IFNs tested, IFN-β was identified to be the most potent cytokine mediating the upregulation of these three substrates. Therefore, this cytokine was subsequently used to test additional viruses. Similar results were obtained using a panel of transmitted/founder (TF) and 6-month (6mo) consensus and chronic (Chr) viruses from clades B and C (Fig. 2). Enhancement of cell surface BST-2, NTB-A, and PVR was observed with all viruses tested upon treatment with IFN-β (Fig. 2A). Again, from the three TM proteins tested, BST-2 remained the only one to be efficiently downregulated by all the viruses (Fig. 2B). In agreement with the results shown in Fig. 1E, a strong positive correlation between BST-2 levels and cell surface NTB-A and PVR was observed (Fig. 2C). Altogether, these results demonstrate that type I IFNs affect the capacity of HIV-1 to downregulate NTB-A and PVR.

10.1128/mBio.01113-19.1 FIG S1 Experimental procedures. Download FIG S1, PDF file, 0.9 MB. Copyright © 2019 Prévost et al. 2019 Prévost et al. This content is distributed under the terms of the Creative Commons Attribution 4.0 International license.

10.1128/mBio.01113-19.2 FIG S2 Role of Vpu and Nef in HIV-1-mediated downregulation of BST-2, NTB-A, and PVR. Download FIG S2, PDF file, 0.2 MB. Copyright © 2019 Prévost et al. 2019 Prévost et al. This content is distributed under the terms of the Creative Commons Attribution 4.0 International license.

### Type I IFNs reduce the capacity of Vpu to downregulate NTB-A and PVR.

**FIG 3:**
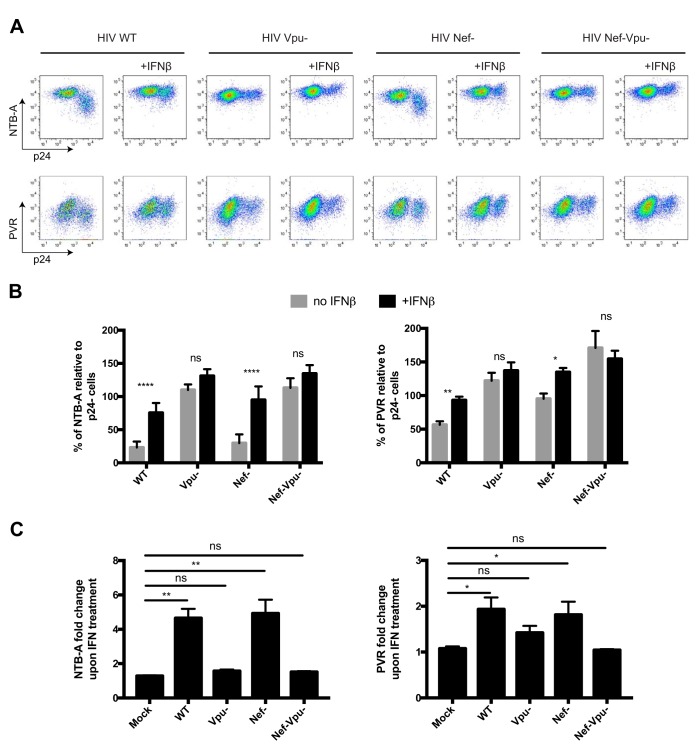
IFN-β impairs Vpu’s ability to downregulate cell surface NTB-A and PVR. Primary CD4^+^ T cells were infected with CH58 TF virus either WT or defective for Nef and/or Vpu expression. At 24 h postinfection, infected cells were either mock treated or treated for 24 h with IFN-β. At 48 h postinfection, cells were stained with anti-NTB-A or anti-PVR Abs, followed by the appropriate secondary Abs. (A) Dot plot depicting representative stainings with or without IFN-β treatment. (B and C) The graphs shown represent the percentage of MFI on the p24^+^ population over the MFI on the p24^−^ population (B) or the fold change in MFI upon IFN treatment on the different populations (C). All graphs shown represent data obtained from at least 5 independent experiments. Error bars indicate means ± SEM. Statistical significance was tested using a multiple *t* test, correcting for multiple comparisons using the Bonferroni-Dunn method (B), and a Kruskal-Wallis test (C) (*, *P* < 0.05; **, *P* < 0.01; ****, *P* < 0.0001; ns, nonsignificant).

Since Vpu is involved in BST-2, NTB-A, and PVR downregulation (Fig. S2), we further investigated the role of this accessory protein in the observed phenotype. Primary CD4^+^ T cells were infected with either wild-type (WT) or *vpu*-defective CH58 TF viruses, and the effect of IFN-β on cell surface expression of NTB-A and PVR was evaluated by flow cytometry. Consistent with the data shown in Fig. 1 and 2, IFN-β significantly enhanced NTB-A and PVR levels on WT-infected cells (Fig. 3A to C). In contrast, no significant increase was observed when cells were infected with the *vpu*-defective virus, suggesting that the effect of IFN-β treatment depends on Vpu expression. The HIV-1 accessory protein Nef contributes to HIV-1-mediated PVR downregulation but not NTB-A or BST-2 downregulation (Fig. S2). To evaluate the effect of IFN-β on Nef-mediated PVR downregulation, we also tested the effect of IFN-β treatment on cells infected with a *nef*-defective variant of CH58 TF. In contrast to Vpu, Nef expression did not alter the capacity of IFN-β to enhance cell surface NTB-A and PVR (Fig. 3A to C). Taken together, these data suggest that IFN-β impairs specifically the ability of Vpu to downregulate the NK cell ligands NTB-A and PVR, but not BST-2.

### BST-2 impairs Vpu’s ability to downregulate cell surface NTB-A and PVR.

**FIG 4:**
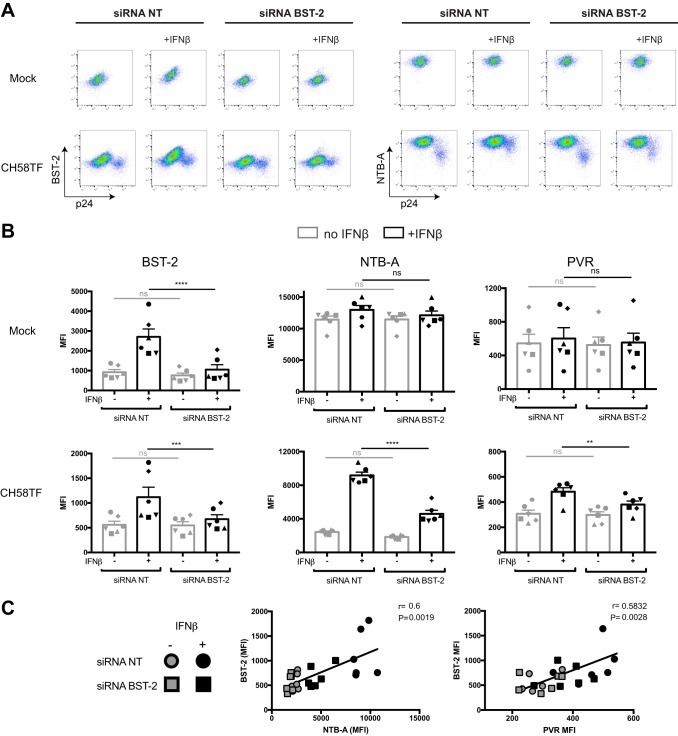
IFN-β enhances cell surface NTB-A and PVR in a BST-2-dependent manner. Primary CD4^+^ T cells were mock infected or infected with CH58 TF WT virus. At 24 h postinfection, infected cells were electroporated with siRNAs targeting BST-2 mRNA or nontargeting siRNAs, followed by treatment (or not) with IFN-β. At 48 h postinfection, cells were stained with anti-BST-2, anti-NTB-A, or anti-PVR Abs, followed by the appropriate secondary Abs. (A) Dot plot depicting representative stainings. (B) The graphs shown represent the MFI obtained on mock-infected or infected (p24^+^) cells in 6 independent experiments. Statistical significance was tested using a paired one-way analysis of variance (**, *P* < 0.01; ***, *P* < 0.001; ****, *P* < 0.0001; ns, nonsignificant). (C) Correlations between the levels of cell surface BST-2 and the levels of NTB-A or PVR on the infected (p24^+^) population upon siRNA electroporation and treatment with IFN-β were tested using Spearman rank correlation test.

Since only BST-2 remains efficiently downregulated by Vpu upon IFN treatment, we explored the role of a Vpu–BST-2 functional interaction in the IFN-induced upregulation of NTB-A and PVR. Primary CD4^+^ T cells, mock infected or infected with HIV-1 CH58 TF, were electroporated with small interfering RNAs (siRNAs) targeting BST-2 mRNA or nontargeting (NT) siRNAs prior to IFN-β treatment. Electroporation with siRNA targeting BST-2 prevented IFN-induced BST-2 upregulation in the context of both mock-infected and CH58 TF-infected cells (Fig. 4A and B). While BST-2 knockdown had no effect on NTB-A and PVR expression in mock-infected cells, it significantly reduced the enhancement of cell surface NTB-A and PVR detected upon type I IFN treatment in infected (p24^+^) cells. This suggests that Vpu partially recovers its ability to downregulate these NK cell ligands upon IFN treatment, provided that BST-2 is depleted. Of note, BST-2 levels and cell surface NTB-A or PVR expression correlated significantly (Fig. 4C). These results demonstrate that Vpu’s ability to downregulate cell surface NTB-A and PVR can be impaired in a BST-2-dependent manner.

**FIG 5:**
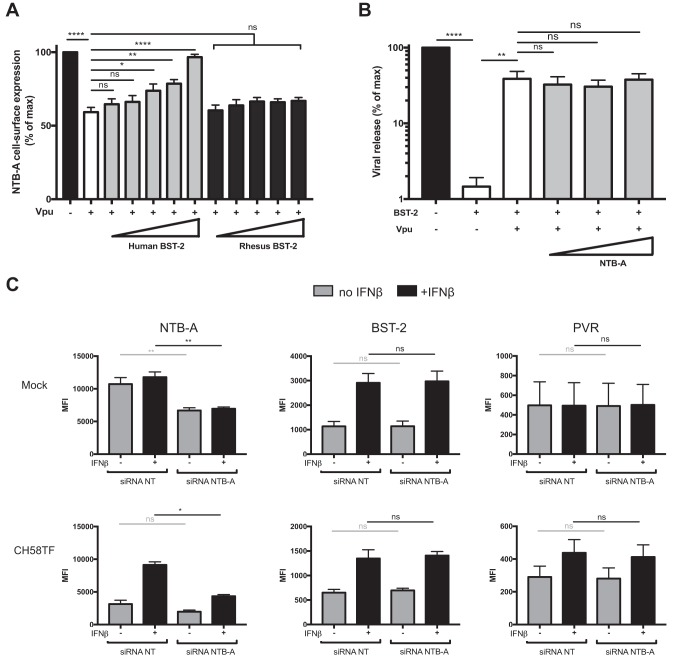
Expression of human BST-2 alone is sufficient to impair the ability of Vpu to downregulate NTB-A. (A) HEK293T cells were cotransfected with plasmids expressing Vpu (40 ng), NTB-A (50 ng), and GFP (150 ng) in the presence of increasing concentration of a human or rhesus macaque BST-2 expression vector (0, 10, 20, 50, 100, 200 ng). At 2 days posttransfection, cells were stained with anti-NTB-A Abs, followed by the appropriate secondary Abs. These data sets are representative of those from 5 independent experiments. (B) HEK293T cells were cotransfected with plasmids harboring a NL4.3 ΔVpu proviral construct (500 ng), human BST-2 (50 ng), and Vpu (40 ng) in the presence of increasing concentrations of the NTB-A expression vector (0, 50, 100, and 200 ng). Cell culture supernatants containing released infectious virions were harvested at 48 h after transfection and assayed for infectivity on TZM-bl cells. These data sets are representative of those from 4 independent experiments. (C) Primary CD4^+^ T cells were mock infected or infected with CH58 TF WT virus. At 24 h postinfection, infected cells were electroporated with siRNAs targeting NTB-A mRNA or nontargeting siRNAs, followed by treatment (or not) with IFN-β. At 48 h postinfection, cells were stained with anti-BST-2, anti-NTB-A, or anti-PVR Abs, followed by the appropriate secondary Abs. The graphs shown represent the MFIs obtained in 3 independent experiments. Statistical significance was tested using an unpaired *t* test or a Mann-Whitney test based on statistical normality (A and B) or a paired one-way analysis of variance (C) (*, *P* < 0.05; **, *P* < 0.01; ****, *P* < 0.0001; ns, nonsignificant).

To evaluate if BST-2 expression is sufficient to impair Vpu’s ability to downregulate NTB-A, we cotransfected HEK293T cells with plasmids coding for Vpu, NTB-A, and human or rhesus macaque BST-2. Rhesus macaque BST-2 cannot be counteracted by HIV-1 Vpu due to key mutations in its TMD (36, 37). In this system, we observed a robust Vpu-mediated NTB-A downregulation (Fig. 5A). In agreement with results obtained with infected primary cells, increasing amounts of human BST-2 but not of rhesus macaque BST-2 progressively abrogated the ability of Vpu to downregulate NTB-A (Fig. 5A). This confirms that BST-2 expression is sufficient to abrogate the capacity of Vpu to target NTB-A, provided that the BST-2 being expressed is susceptible to Vpu.

To evaluate if this phenotype was specific to BST-2 expression, we determined whether enhanced expression of NTB-A affected the ability of Vpu to counteract BST-2 and enhance viral release. As expected, BST-2 expression potently reduced HIV-1 particle release, and this was partially overcome by Vpu (Fig. 5B). In contrast to the impact of BST-2 expression on Vpu-mediated NTB-A downregulation, increasing amounts of NTB-A did not affect the capacity of Vpu to enhance viral release upon BST-2 expression (Fig. 5B). Consistent with these findings, electroporation of siRNA against NTB-A did not enhance the capacity of Vpu to control BST-2 or PVR cell surface levels in the absence or presence of type I IFNs (Fig. 5C). These observations support a model where Vpu preferentially targets BST-2 over NTB-A.

### BST-2 upregulation by type I IFN affects the ability of Vpu to downmodulate CD62L but not CD4.

**FIG 6:**
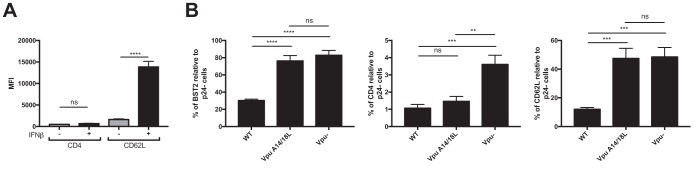
Type I IFNs enhance cell surface CD62L but not CD4 on HIV-1-infected cells. (A) Primary CD4^+^ T cells were infected with CH58 TF virus and either mock treated or treated for 24 h with IFN-β. At 48 h postinfection, cells were stained with anti-CD4 or anti-CD62L Abs, followed by the appropriate secondary Abs. The graph shown represents the median fluorescence intensities (MFI) detected on p24^+^ cells. (B) Primary CD4^+^ T cells were infected with CH58 TF virus expressing the Vpu WT or Vpu A14/18L or CH58 TF virus defective for Vpu expression (Vpu^−^). At 48 h postinfection, cells were stained with anti-BST-2, anti-CD4, or anti-CD62L Abs, followed by the appropriate secondary Abs. The graphs shown represent the percentage of BST-2, CD4, and CD62L detected on the p24^+^ cells relative to the p24^−^ cells. All these graphs represent data obtained in at least 6 independent experiments. Error bars indicate means ± SEM. Statistical significance was tested using an unpaired *t* test or the Mann-Whitney test based on statistical normality (**, *P* < 0.01; ***, *P* < 0.001; ****, *P* < 0.0001; ns, nonsignificant).

**FIG 7:**
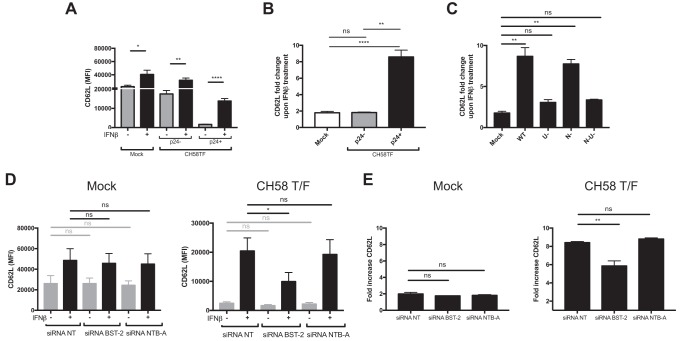
Type I IFN-induced BST-2 upregulation prevents the capacity of Vpu to downregulate CD62L. (A to C) Primary CD4^+^ T cells were infected with CH58 TF virus, either WT or defective for Nef and/or Vpu expression. At 24 h postinfection, infected cells were either mock treated or treated for 24 h with IFN-β. At 48 h postinfection, cells were stained with anti-CD62L Abs, followed by the appropriate secondary Abs. (A to C) The graphs shown represent the median fluorescence intensities (MFI) (A), the fold change in MFI upon IFN treatment detected on mock-infected, p24^−^, and p24^+^ cells in the context of WT infection (B), and the fold change in MFI upon IFN treatment detected on p24^+^ cells in the context of infection with WT virus and virus lacking Vpu (U^−^), Nef (N^−^), and both Nef and Vpu (N^−^U^−^) (C). (D and E) Primary CD4^+^ T cells were mock infected or infected with CH58 TF WT virus. At 24 h postinfection, cells were electroporated with siRNAs targeting BST-2 or NTB-A mRNA or nontargeting siRNAs, followed by treatment (or not) with IFN-β. At 48 h postinfection, cells were stained with anti-CD62L Abs, followed by the appropriate secondary Abs. The graphs shown represent the median fluorescence intensities (MFI) (D) or the fold change in MFI upon IFN treatment detected on mock-infected or p24^+^ virus-infected cells (E). All these graphs represent data obtained in at least 3 independent experiments. Error bars indicate means ± SEM. Statistical significance was tested using an unpaired *t* test or the Mann-Whitney test based on statistical normality (A and B), a Kruskal-Wallis test (C), or a paired one-way analysis of variance (D and E) (*, *P* < 0.05; **, *P* < 0.01; ****, *P* < 0.0001; ns, nonsignificant).

To confirm that BST-2 expression affects the ability of Vpu to downregulate substrates that depend on its TMD, we evaluated the effect of IFN-β treatment on two other Vpu substrates: CD4 and CD62L. Consistent with the involvement of the cytoplasmic domain of Vpu for CD4 downregulation and not its TMD (38), CD4 levels on HIV-1-infected primary CD4^+^ T cells were not affected by IFN-β treatment (Fig. 6A) even in the absence of Nef (Fig. S3). In contrast, IFN-β treatment significantly enhanced the cell surface levels of CD62L (Fig. 6A). As expected, mutation of Vpu’s TMD (A14L/A18L) abrogated Vpu’s capacity to downregulate CD62L but not CD4, confirming the requirement of Vpu’s TMD for Vpu-mediated CD62L downregulation (Fig. 6B). We then characterized CD62L expression in mock-infected, bystander, and infected primary CD4^+^ T cells. We found that IFN-β enhanced CD62L expression on all these populations but did so more significantly on the infected cell population (Fig. 7A and B). Similar to what we observed for NTB-A and PVR, IFN-β specifically impaired the ability of Vpu to downregulate CD62L, and this activity was significantly enhanced by BST-2 depletion (Fig. 7C to E). In summary, these results suggest that occupation of Vpu’s TMD by BST-2 affects Vpu’s capacity to target multiple substrates.

10.1128/mBio.01113-19.3 FIG S3 Effect of IFN-β on cell surface CD4 levels. Download FIG S3, PDF file, 0.1 MB. Copyright © 2019 Prévost et al. 2019 Prévost et al. This content is distributed under the terms of the Creative Commons Attribution 4.0 International license.

### IFN-β sensitizes HIV-1-infected cells to NK cell responses in an NTB-A- and DNAM-1-dependent manner.

**FIG 8:**
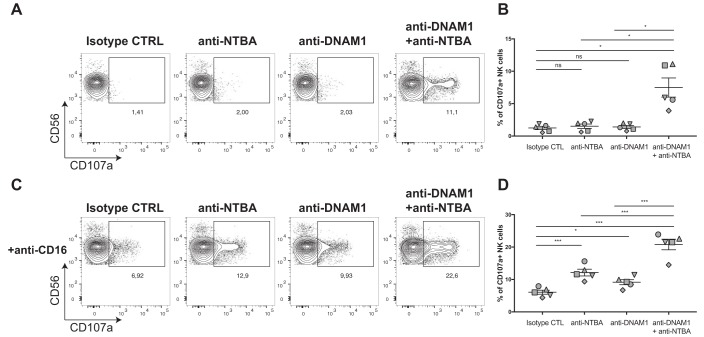
Stimulation of NK cells via NTB-A and DNAM-1 induces direct and Ab-dependent NK cell responses. (A and B) P815 cells were incubated with anti-NTB-A Abs and/or anti-DNAM-1 Abs or a matched IgG isotype control prior to being mixed with purified NK cells and incubated for 4 h. (C and D) Alternatively, P815 cells were also incubated with anti-CD16 Abs. CD3^−^ CD56^+^ cells were evaluated for the percentage of cell surface CD107a expression. (A and C) Dot plots depict representative NK cell stimulation. (B and D) The graphs shown represent the percentage of CD107a expression among CD3^−^ CD56^+^ NK cells obtained in 5 independent experiments. Statistical significance was tested using a paired *t* test (*, *P* < 0.05; ***, *P* < 0.001; ns, nonsignificant). CTRL and CTL, control.

To evaluate the functional consequences of type I IFN-mediated impairment of NTB-A and PVR downregulation, we characterized their contribution in stimulating NK cell cytotoxic activity using a redirection degranulation assay. Fc gamma receptor-positive (FcγR^+^) P815 cells were incubated with antibodies known to engage NTB-A or DNAM-1 or with their matched IgG isotypes. The cells were then incubated with human primary NK cells. In this assay, the Fc portion of the Ab interacts with the FcγR present on P815 target cells, thus allowing NK cell activation through a single receptor or a combination of receptors (here, NTB-A and/or DNAM-1). NK cell degranulation was monitored by detection of cell surface CD107a. Consistent with previous reports (39), the stimulation of either NTB-A or DNAM-1 receptors alone was not sufficient to trigger NK cell degranulation (Fig. 8A). However, the combination of NTB-A and DNAM-1 Abs resulted in a significant increase in NK cell degranulation (Fig. 8A and B), indicating that there is a functional interplay between these receptors to stimulate NK cells. Accordingly, we found that these receptors also positively modulated NK-mediated antibody-dependent cellular cytotoxicity (ADCC) (Fig. 8C and D). Even though stimulation through CD16 was sufficient to induce NK cell degranulation, addition of an anti-NTB-A Ab, an anti-DNAM-1 Ab, or both increased significantly the magnitude of NK cell degranulation (Fig. 8C and D). Altogether, these results indicate that the stimulation of NK cells via DNAM-1 and NTB-A is sufficient to trigger both direct and Ab-dependent NK cell responses.

**FIG 9:**
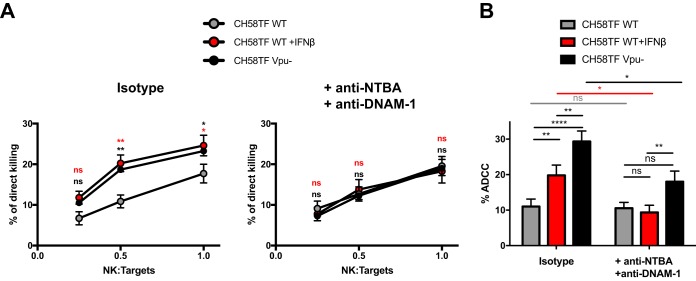
Type I IFNs sensitize HIV-1-infected cells to NK cell responses in an NTB-A- and DNAM-1-dependent manner. (A) Primary CD4^+^ T cells infected with CH58 TF WT virus (treated or not with IFN-β) or its *vpu*-defective counterpart (Vpu^−^) were used as target cells, while autologous NK cells were used as effector cells to perform a direct killing assay. NK cells were preincubated in the presence of anti-NTB-A and anti-DNAM-1 or their matched IgG isotype controls prior to incubation with target cells for 5 h at different NK cell/target cell ratios (1:4, 1:2, 1:1). The graphs shown represent the percentages of direct killing obtained from 3 independent experiments done in triplicate. (B) Alternatively, autologous PBMCs were used as effector cells in a well-established FACS-based ADCC assay. Autologous PBMCs were preincubated in the presence of anti-NTB-A and anti-DNAM-1 or their matched IgG isotype controls prior to incubation with target cells for 5 h. The graph shown represents the percentages of ADCC obtained with bNAb 3BNC117 in the presence or absence of blocking Abs. Statistical significance was tested using an unpaired *t* test (*, *P* < 0.05; **, *P* < 0.01; ****, *P* < 0.0001; ns, nonsignificant).

The potential contribution of NTB-A and PVR in HIV-1-infected cell susceptibility to NK cells was evaluated using a direct killing assay. Primary CD4^+^ T cells infected with CH58 TF WT virus or its *vpu*-defective counterpart were used as targets in the presence of increasing amounts of autologous primary NK cells. Confirming the protective effect of Vpu expression on NK cell-mediated killing (26, 27), WT-infected cells were less susceptible to autologous NK cell responses than cells infected with a *vpu*-defective virus (Fig. 9A). Interestingly, treatment of WT-infected cells with IFN-β significantly increased their susceptibility to autologous NK cell lysis (Fig. 9A) to levels similar to those reached with *vpu*-defective virus-infected cells. Strikingly, this increase in direct NK cell lysis could be abrogated when NK cells were preincubated with a combination of blocking antibodies against the NK cell receptors NTB-A and DNAM-1 (Fig. 9A). Similarly, the capacity of 3BNC117, a broadly neutralizing antibody (bNAb; targeting the CD4-binding site), to mediate ADCC against HIV-1-infected cells was significantly enhanced upon IFN-β treatment (Fig. 9B). IFN-treated infected cells remained less susceptible to ADCC than cells infected with a *vpu*-defective virus. This is consistent with the capacity of the WT virus to downregulate BST-2 despite IFN treatment (Fig. 1A and B). BST-2 downregulation facilitates viral release, thus decreasing the accumulation of Env at the cell surface (Fig. S4). However, the ADCC responses directed against WT-infected cells treated with IFN or cells infected with a *vpu*-defective virus were significantly decreased by the addition of blocking antibodies against NTB-A and DNAM-1 (Fig. 9B). Taken together, these results reveal that IFN-β enhances the susceptibility of HIV-1-infected cells to autologous NK cell responses in an NTB-A- and DNAM-1-dependent manner.

10.1128/mBio.01113-19.4 FIG S4 Recognition of HIV-1-infected cells by bNAb 3BNC117 upon treatment with IFN-β. Download FIG S4, PDF file, 0.2 MB. Copyright © 2019 Prévost et al. 2019 Prévost et al. This content is distributed under the terms of the Creative Commons Attribution 4.0 International license.

## DISCUSSION

Vpu is known to downregulate several membrane proteins from the cell surface of HIV-1-infected CD4^+^ T cells. The TMD of Vpu has been shown to be required to downregulate several of them, including BST-2 (9–11), NTB-A (27), PVR (34), and CD62L (Fig. 6). How Vpu manages to target these different substrates using the same domain has not been established. Here we provide the first evidence that under physiologically relevant conditions, BST-2 is preferentially downregulated by Vpu over its other TMD substrates. Specifically, we show that type I IFNs decrease Vpu’s polyfunctionality by upregulating BST-2, thus reducing its capacity to downmodulate the NK cell ligands NTB-A and PVR. Importantly, we show that type I IFNs efficiently sensitize HIV-1-infected cells to both direct and antibody-dependent NK cell responses.

Our results support a model in which the specific occupancy of Vpu’s TMD by BST-2 prevents its ability to target other TM proteins. Consistent with this model, Vpu’s ability to downregulate CD4 was not affected by IFN-β treatment. This activity of Vpu involves the cytoplasmic domain of the viral protein rather than its TMD (38) (Fig. 6B). Consequently, the capacity of Vpu to target CD4 is unlikely to be impacted by the occupation of its TMD by BST-2. While studies have suggested that the Vpu–BST-2 interaction could occur within post-endoplasmic reticulum membranes (40), we cannot rule out the possibility that Vpu could also interact with CD4 and BST-2 in a successive manner. However, supporting our model, we found that the IFN-induced upregulation of BST-2 affected the ability of Vpu to downmodulate another substrate that depends on the integrity of Vpu’s TMD: the CD62L homing receptor (Fig. 6 and 7). Our findings therefore raise the intriguing possibility that numerous Vpu functions relying on its TMD could be affected by the upregulation of BST-2. One distinctive feature of group M Vpu proteins is their capacity to target BST-2 to an ESCRT-dependent endosomal degradation pathway. However, increasing evidence suggests that BST-2 counteraction by Vpu results from the subversion of BST-2 trafficking prior to its degradation. While BST-2 degradation by Vpu does not appear to be required to counteract BST-2 (11, 14, 15, 18, 19, 22), it could represent an effective mechanism to unleash Vpu’s TMD to interact with other cellular substrates. One could speculate that Vpu’s polyfunctionality could therefore rely on its ability to degrade BST-2.

Our results provide the first indication that that there is a hierarchy of Vpu targets under type I IFN responses. In contrast to NTB-A, PVR, and CD62L, both CD4 and BST-2 remain efficiently targeted by Vpu despite IFN treatment, which further supports the notion that these two proteins represent the major targets of Vpu (Fig. 1, 2, and 6). BST-2 counteraction and CD4 downregulation were found to be critical for viral egress and immune evasion. Vpu antagonism of BST-2 plays a crucial role in enhancing virus replication and release in human CD4^+^ T cells, particularly in the presence of IFN (41). This activity of Vpu was recently identified to be a major determinant of HIV-1 IFN resistance (41). CD4 degradation by Vpu also participates in the release of fully infectious viral particles by preventing the intracellular interaction between CD4 and Env (24, 25). Finally, both of Vpu’s activities contribute to protect HIV-1-infected cells from ADCC responses by preventing the exposure of ADCC-mediating Env epitopes (42).

The reason for this preference of Vpu for BST-2 over NTB-A and PVR remains to be determined. One can speculate that Vpu’s TMD could have a higher affinity for BST-2’s TMD than for NTB-A’s or PVR’s TMD. A recent study by Cole et al. characterized the heterooligomer formation between the TMD of Vpu and its substrates in a lipid environment using Förster resonance energy transfer (FRET) (43). Data from this study show that peptides of Vpu’s TMD have a lower affinity for TMD peptides of NTB-A, PVR, and BST-2 than for known TM helix dimers (43). The interaction between Vpu and dimeric BST-2, however, remained more energetically favorable than Vpu–NTB-A and Vpu-PVR interactions, potentially due to the additive effects of multiple TMD-TMD interactions (43). BST-2 forms disulfide-linked dimers, and BST-2 dimerization is required for its antiviral function (7, 44–46). The preference of Vpu for BST-2 could therefore result from its higher affinity for BST-2 dimers than for other monomeric substrates. In the study of Cole et al., PVR and NTB-A TMDs were found to compete with BST-2 TMD for the binding of the Vpu TMD peptide in a lipid environment (43). It is possible that this reduced Vpu–BST-2 interaction detected in the presence of NTB-A’s or PVR’s TMD is not sufficient to have a significant impact on Vpu function. This apparent contradiction could be explained if the affinity of TMD peptides artificially embedded in a lipid environment did not recapitulate the affinity and/or function of this domain expressed within the full protein. In that regard, our results using infected primary CD4^+^ T cells or Vpu-expressing cells revealed that NTB-A expression has no effect on the capacity of Vpu to antagonize BST-2 (Fig. 5B and C). Inversely, BST-2 expression greatly reduced the capacity of Vpu to downregulate NTB-A and PVR (Fig. 4 and 5).

HIV/simian immunodeficiency virus infection is followed by an intense cytokine storm involving type I IFNs. An intact IFN response during the acute phase of infection appears to be crucial for viral control (47). While BST-2 expression is upregulated upon HIV-1 infection by endogenous type I IFNs, a recent study revealed that basal BST-2 levels are sufficient to suppress HIV-1 replication (48). The counteraction of BST-2 by Vpu consequently confers a selective advantage for viral spread *in vivo*, at least in humanized mice (48–50). Our observation that Vpu preferentially targets BST-2 over other TM proteins (NTB-A, PVR, and CD62L) upon IFN treatment raises the possibility that the polyfunctionality of Vpu could be reduced during acute HIV-1 infection. The upregulation of BST-2 observed during this stage of infection (47, 48) could impact the capacity of Vpu to target multiple TM proteins. This could explain why studies using humanized mouse models of acute HIV-1 infection failed to detect the Vpu-mediated downregulation of NTB-A *in vivo*, despite detecting it *in vitro* (49, 50). In these mouse models, strong type I IFN responses and subsequent BST-2 upregulation were detected upon HIV-1 infection (48). It is then conceivable that the capacity of Vpu to target NTB-A *in vivo* could have been impacted by type I IFN-mediated BST-2 upregulation. Resistance to type 1 IFNs represents a key determinant of HIV-1 transmission fitness. Transmitted/founder (TF) viruses are phenotypically distinct, and increased IFN resistance represents their most distinguishing property (41, 51–54). However, resistance to IFNs is not static during the course of HIV-1 infection. Previous studies revealed that IFN resistance declines rapidly within the first 6 months of infection (53, 54) but then tends to increase again at later stages of disease progression (53). In this study, we found that type I IFNs affect the downregulation of NTB-A and PVR by HIV-1, including by viruses that differ in their sensitivity to IFNs (Fig. 2). All tested viruses, including TF, 6-month, and chronic viruses, were found to be sensitive, at different levels, to this IFN activity. This suggests that type I IFNs could differentially affect Vpu polyfunctionality at different stages of infection. Future studies using longitudinally linked viruses are needed to determine whether the capacity of Vpu to downmodulate NTB-A and PVR upon IFN treatment varies during the course of infection.

We also found that type I IFNs enhance the susceptibility of HIV-1-infected cells to NK cell responses. We demonstrated that stimulation of human NK cells via the NTB-A and DNAM-1 receptors is sufficient to induce NK cell degranulation. We also provide evidence that NTB-A and DNAM-1 are critical players for NK cell-mediated ADCC. We found that there is a functional interplay between these receptors together with CD16 to enhance NK cell degranulation, suggesting that they act as coreceptors of CD16. By preventing Vpu’s ability to downregulate NTB-A and PVR, type I IFNs impair Vpu’s capacity to protect infected cells from NK responses. Importantly, we found that type I IFNs efficiently sensitize cells infected with an IFN-resistant TF virus (CH58 TF) (41, 53, 54) to autologous NK cell lysis. This suggests that, for the virus, targeting BST-2 as part of its resistance to type I IFNs takes precedence over protecting HIV-1-infected cells from NK cell responses, at least early in infection, when type I IFN responses play an important role in systemic viral control.

Our findings further support the idea that type I IFNs could be utilized to harness NK cell responses against HIV-1-infected cells. Stimulation with IFN-α was found to augment NK cell cytokine secretion, polyfunctionality, and degranulation, as well as the capacity of NK cells to lyse autologous HIV-1-infected cells (55–57). Additionally, type I IFNs enhance the susceptibility of HIV-1-infected cells to ADCC responses by increasing the level of ADCC-mediating Env epitopes on infected cells (35, 58). Now we demonstrate that this increase in ADCC responses also depends on the capacity of type I IFNs to prevent Vpu-mediated NTB-A and PVR downmodulation (Fig. 9B). Altogether, this suggests that type I IFNs could both directly enhance NK cell effector function and also augment the susceptibility of infected cells to NK cells. Interestingly, administration of IFN-α during chronic HIV-1 infection was found to enhance BST-2 expression and tended to decrease HIV-1 replication (59–61). Studies also revealed that treatment with IFN-α leads to a moderate but sustained decline of integrated HIV-1 DNA in CD4^+^ T cells (60, 62). While all studies to date in chronic infection have used IFN-α2a, other IFN-α subtypes, as well as IFN-β, have been shown to be more potent at upregulating BST-2 and restricting HIV-1 infection (Fig. 1) (52, 63, 64). Consequently, whether these cytokines would have a more pronounced effect on viral replication and the clearance of the viral reservoir *in vivo* remains to be determined.

In summary, we demonstrate that type I IFN-mediated upregulation of BST-2 greatly impairs Vpu’s polyfunctionality. Upon type I IFN treatment, Vpu preferentially targets BST-2 over the NK cell ligands NTB-A and PVR, thus reducing its ability to protect HIV-1-infected cells from NK cell responses.

## MATERIALS AND METHODS

### Ethics statement.

Written informed consent was obtained from all study participants, and the research adhered to the ethical guidelines of CRCHUM and was reviewed and approved by the CRCHUM Institutional Review Board (Ethics Committee approval number CE 16.164-CA). The research adhered to the standards indicated by the Declaration of Helsinki. All participants were adults and provided informed written consent prior to enrollment, in accordance with Institutional Review Board approval.

### Cell culture and isolation of primary cells.

HEK293T human embryonic kidney cells and P815 mouse lymphoblast-like mastocytoma cells (obtained from ATCC) were grown as previously described (39, 65). Primary human peripheral blood mononuclear cells (PBMCs), CD4^+^ T cells, and NK cells were isolated, activated, and cultured as previously described (66, 67). Briefly, PBMCs were obtained by Ficoll density gradient from whole-blood samples obtained from 9 different HIV-1-negative donors. CD4^+^ T lymphocytes and NK cells were purified from resting PBMCs by negative selection using immunomagnetic beads per the manufacturer’s instructions (StemCell Technologies, Vancouver, BC, Canada). CD4^+^ T cells were activated with phytohemagglutinin-L (10 μg/ml) for 48 h and then maintained in RPMI 1640 complete medium supplemented with recombinant interleukin-2 (100 U/ml; R&D Systems) (see Fig. S1 in the supplemental material). NK cells were isolated and rested overnight in RPMI 1640 complete medium on the day prior to the redirection and killing assays.

### Proviral constructs and plasmids.

Transmitted/founder (TF) viruses and the corresponding 6-month (6mo) consensus and chronic (Chr) infectious molecular clones (IMCs) of patients CH40, CH58, CH77, CH167, CH198, CH236, CH470, CH505, CH850, and STCO were inferred, constructed, and biologically characterized (51, 53, 68–71). CH58 IMCs defective for Vpu and/or Nef expression or harboring the TMD mutations A14L/A18L, as well as the NL4.3 proviral construct defective for Vpu expression, were previously described (41, 72, 73). The plasmids expressing hemagglutinin (HA)-tagged Vpu 2_87 (pCR3.1-Vpu2_87-HA), human BST-2 (pCR3.1-human Tetherin), rhesus macaque BST-2 (pCR3.1-rhesus Tetherin), NTB-A (pQCXIP_human SLAM6/NTB-A), or green fluorescent protein (GFP) (pCR3.1-GFP) were previously described (14, 36, 74).

### Viral production and infections.

To achieve similar levels of infection among all viruses tested, vesicular stomatitis virus G (VSV-G)-pseudotyped HIV-1 isolates were produced in HEK293T cells and titrated in activated primary CD4^+^ T cells to achieve a 10 to 20% infection. Viruses were then used to infect activated primary CD4^+^ T cells from healthy HIV-1-negative donors by spin infection at 800 × *g* for 1 h in 96-well plates at 25°C. All experiments using VSV-G-pseudotyped HIV-1 isolates were done in a biosafety level 3 laboratory following manipulation protocols accepted by the CRCHUM Biosafety Committee, which respects the requirements of the Public Health Agency of Canada.

### Antibodies.

A detailed list of the Abs used for cell surface staining and measurement of NK cell responses is presented in Text S1 in the supplemental material.

10.1128/mBio.01113-19.5 TEXT S1 Supplemental methods. Download Text S1, DOCX file, 0.02 MB. Copyright © 2019 Prévost et al. 2019 Prévost et al. This content is distributed under the terms of the Creative Commons Attribution 4.0 International license.

### Flow cytometry analysis of cell surface staining.

Cell surface stainings were performed as previously described (66) and detailed in Text S1 in the supplemental material. The percentage of BST-2, NTB-A, and PVR levels detected on infected p24^+^ cells relative to uninfected p24^−^ cells was calculated with the following formula: (MFI detected on p24^+^ cells/MFI detected on p24^−^ cells) × 100, where MFI represents the mean fluorescence intensity.

### Type I IFN treatments.

Type I IFNs were titrated to identify the optimal concentrations that efficiently enhanced cell surface BST-2 levels on HIV-1-infected primary CD4^+^ T cells. IFN-β (Rebif; EMD Serono Inc.) (52) was added to the cells at 1 ng/ml. All different IFN-α types tested (PBL Assay Science) were reconstituted in RPMI 1640 complete medium at 1 × 10^7^ U/ml, aliquoted, and stored at −80°C. IFN-α was then added to the cells at 1,000 U/ml. Type I IFNs were added to the cells at 24 h postinfection, 24 h before cell surface staining or killing assays.

### siRNA electroporation.

Primary CD4^+^ T cells mock infected or infected with CH58 TF were electroporated with pools of 4 siRNAs to silence BST-2 (ON-TARGETplus Human BST-2 siRNA-SMART pool; Dharmacon) or NTB-A (ON-TARGETplus Human SLAMF6 siRNA-SMART pool; Dharmacon) expression. Briefly, at 24 h postinfection, infected or mock-infected primary CD4^+^ T cells were resuspended at a concentration of 5 × 10^7^ cells/ml in Opti-MEM medium (Invitrogen), and 55 μl of the cell suspension was transferred into a 1-mm electroporation cuvette (Harvard Apparatus). Pools of nontargeting (NT) siRNA or siRNA targeting BST-2 or NTB-A sequences were then added to the cells (150 pmol/3 × 10^6^ cells). Further, cells were electroporated at 250 V for 2 ms using a BTX Gemini X2 electroporation system (Harvard Apparatus). Subsequently, electroporated cells were resuspended in RPMI 1640 complete medium and were treated or not with IFN-β (1 ng/ml).

### BST-2/NTB-A competition assay.

HEK293T cells were seeded at 1.5 × 10^5^ cells per well of a 24-well plate the day before transfection. Cells were cotransfected with 50 ng of an NTB-A-expressing plasmid (pQCXIP_human SLAM6/NTB-A), 40 ng of an Vpu-expressing plasmid (pCR3.1-Vpu2_87-HA), and 150 ng of a GFP-expressing plasmid (pCR3.1-GFP) in the presence of increasing concentrations of human or rhesus macaque BST-2-expressing plasmids (pCR3.1-human Tetherin or pCR3.1-rhesus Tetherin) (0, 10, 20, 50, 100 or 200 ng) using the Lipofectamine 2000 reagent (Invitrogen). At 2 days posttransfection, cell surface NTB-A levels were monitored by flow cytometry as described above. The fluorescence of stained cells was detected by two-color flow cytometry, and Vpu-mediated NTB-A downmodulation was calculated as described previously (74). Briefly, the mean fluorescence intensities were determined for cells showing specific ranges of GFP expression. The fluorescence values obtained for cells cotransfected with the NTB-A, Vpu, and GFP expressors with or without the human or rhesus macaque BST-2 expressors were compared with the corresponding values for number obtained for cells cotransfected with the NTB-A and GFP expressors to determine the efficiency of NTB-A downmodulation by Vpu. The same GFP gating was used in all calculations.

### Viral release assay.

Subconfluent HEK293T cells were plated in 24-well plates and transfected with NL4.3 ΔVpu proviral construct (500 ng) with or without human the BST-2-expressing plasmid (50 ng; pCR3.1-human Tetherin) in the presence of the Vpu-expressing plasmid (50 ng; pCR3.1-Vpu2_87-HA) and increasing concentrations of the NTB-A-expressing plasmid (0, 50, 100, and 200 ng; pQCXIP_human SLAM6/NTB-A) using 2 μl the Lipofectamine 2000 reagent (Thermo Fisher Scientific). The medium was replaced at 16 h posttransfection, and the supernatants were harvested at 24 h after the medium change and filtered. The infectivity of viral supernatants was determined by infecting TZM-bl cells, and β-galactosidase activity was assayed as previously described (14).

### Measurement of NK cell responses.

Direct and antibody-dependent NK cell responses were measured using the NK cell redirection degranulation assay, the NK cell direct killing assay, and the fluorescence-activated cell sorting (FACS)-based ADCC assay as described in detail in Text S1 in the supplemental material.

### Statistical analysis.

Statistics were analyzed using GraphPad Prism (version 6.01) software (GraphPad, San Diego, CA, USA). Every data set was tested for statistical normality, and this information was used to apply the appropriate (parametric or nonparametric) statistical test. *P* values of <0.05 were considered significant; significance values are indicated in the figure legends.
