# Supplementary material for: Upregulation of BST-2 by Type I Interferons Reduces the Capacity of Vpu To Protect HIV-1-Infected Cells from NK Cell Responses
Source: mBio. 2019 Jun 18;10(3):e01113-19. doi: 10.1128/mBio.01113-19 (PMC6581860; doi:10.1128/mBio.01113-19)
Supplement: FIG S1 [file mBio.01113-19-sf001.pdf]

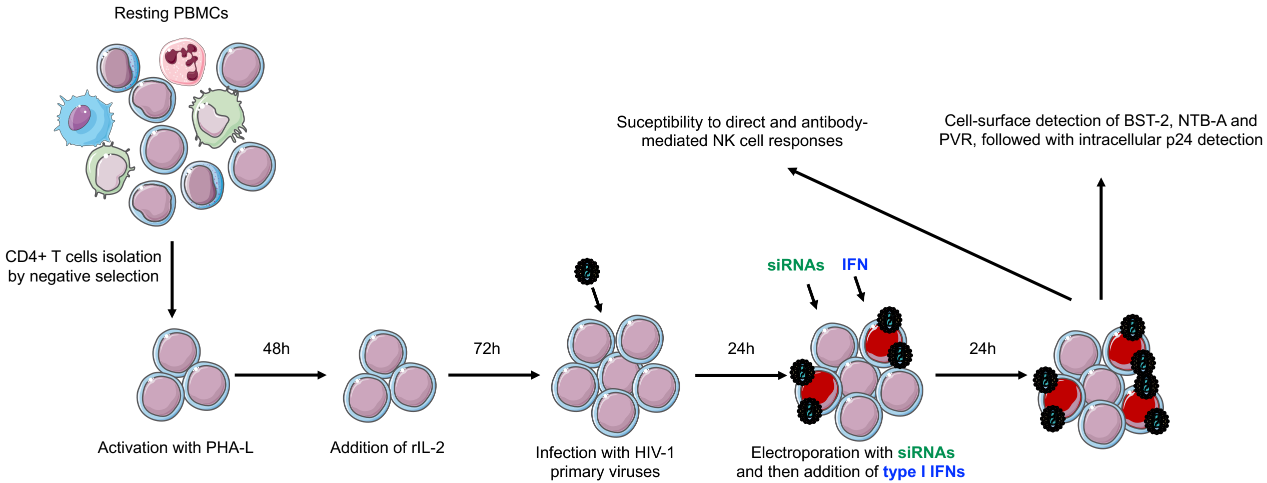

Resting PBMCs
CD4+ T cells isolation by negative selection
Activation with PHA-L
48h
Addition of rIL-2
72h
Infection with HIV-1 primary viruses
24h
siRNAs
IFN
Electroporation with siRNAs and then addition of type I IFNs
24h
Suceptibility to direct and antibody-mediated NK cell responses
Cell-surface detection of BST-2, NTB-A and PVR, followed with intracellular p24 detection
