# Supplementary figures and images for: Upregulation of BST-2 by Type I Interferons Reduces the Capacity of Vpu To Protect HIV-1-Infected Cells from NK Cell Responses
Source: mBio. 2019 Jun 18;10(3):e01113-19. doi: 10.1128/mBio.01113-19 (PMC6581860; doi:10.1128/mBio.01113-19)

**BST-2**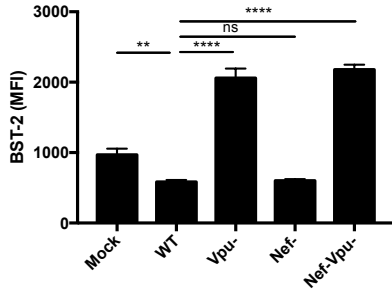**NTB-A**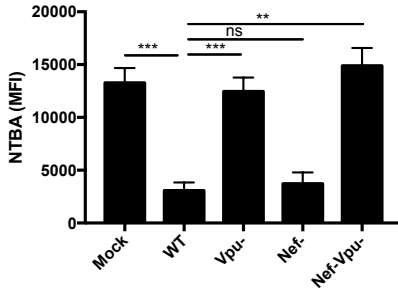**PVR**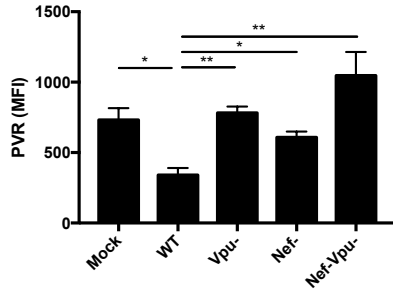

Supplement: FIG S2 [file mBio.01113-19-sf002.pdf]

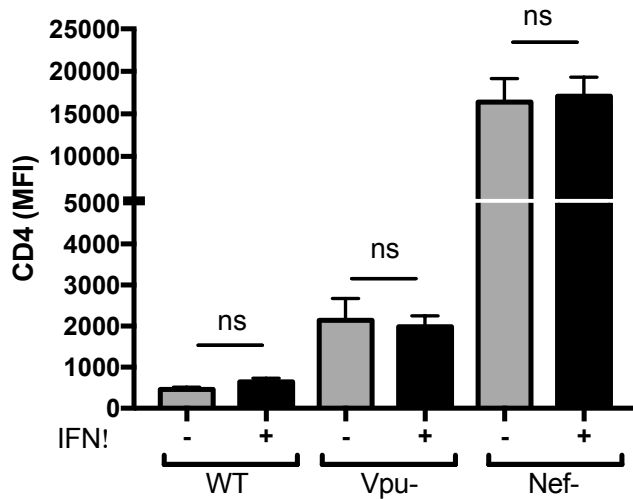

Supplement: FIG S3 [file mBio.01113-19-sf003.pdf]

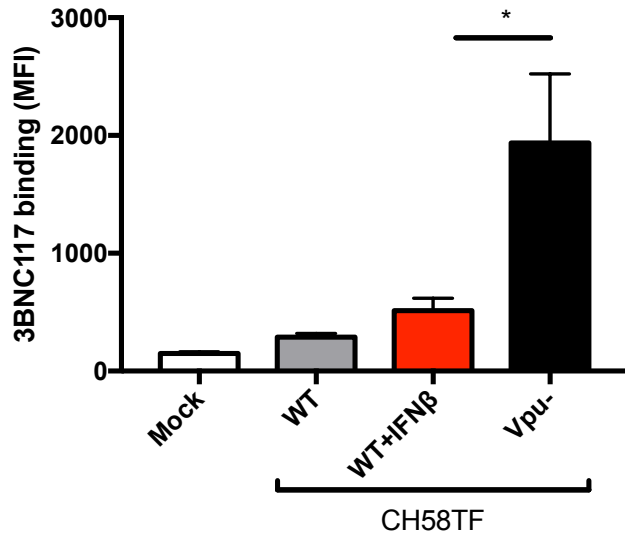

Supplement: FIG S4 [file mBio.01113-19-sf004.pdf]
