# Supplementary material for: Upregulation of BST-2 by Type I Interferons Reduces the Capacity of Vpu To Protect HIV-1-Infected Cells from NK Cell Responses
Source: mBio. 2019 Jun 18;10(3):e01113-19. doi: 10.1128/mBio.01113-19 (PMC6581860; doi:10.1128/mBio.01113-19)
Supplement: TEXT S1 [file mBio.01113-19-s0001.docx]

**SUPPLEMENTAL METHODS**

**Flow cytometry analysis of cell-surface staining**

Binding of antibodies to cell-surface NTB-A (10µg/mL), PVR (10µg/mL), rabbit polyclonal anti-BST-2 (1:2000 dilution), CD62L (1µg/mL), CD4 (1 µg/mL) and anti-HIV-1 Env mAb 3BNC117 (0,5 µg/mL) was performed at 48h post-infection. Infected cells were stained intracellularly for HIV-1 p24, using the Cytofix/Cytoperm Fixation/ Permeabilization Kit (BD Biosciences, Mississauga, ON, Canada) and the fluorescent anti-p24 mAb (PE-conjugated anti-p24, clone KC57; Beckman Coulter/Immunotech). The percentage of infected cells (p24+) was determined by gating the living cell population using a viability dye staining (Aqua Vivid, Thermo Fisher Scientific). Samples were acquired on an LSRII cytometer (BD Biosciences), and data analysis was performed using FlowJo vX.0.7 (Tree Star, Ashland, OR, USA).

**Antibodies**

The following Abs were used as primary Abs for cell-surface staining: mouse anti-human CD352 (NTB-A) (clone NT-7, Biolegend), mouse anti-human CD155 (PVR) (clone SKII.4, Biolegend), rabbit polyclonal anti-human BST2 antiserum (NIH AIDS Reagent Program), mouse anti-CD4 (clone OKT4, eBioscience), mouse anti-CD62L (clone LT-TD180, Invitrogen), anti-HIV-1 Env mAb 3BNC117 (NIH AIDS Reagent Program). Goat anti-mouse and anti-human antibodies pre-coupled to Alexa Fluor 647 (Invitrogen) and BrillantViolet 421-conjugated donkey anti-rabbit antibodies (Biolegend) were used as secondary antibodies in flow cytometry experiments. The following Abs were used for redirection assays and/or killing blockade experiments: PE-anti-human CD56 (clone NCAM16.2, BD Biosciences), APC-anti-human CD107a (clone H4A3, BD Biosciences), BUV395-anti-human CD3 (clone UCHT1, BD Biosciences), mouse anti-human CD352 (NTB-A) (clone NT-7, Biolegend), mouse anti-human CD226 (clone DX11, BD Pharmingen), mouse anti-human CD16 (clone 3G8, Biolegend) and their matched IgG isotype control (clone MOPC-21, Biolegend).

**Redirection assays**

P815 cells were incubated with 5 μg/ml of purified anti-NTB-A Abs and/or anti-DNAM-1 Abs or their matched IgG isotype control Abs, in the presence or not of 0.5 μg/ml of anti-CD16 Abs, for 30 min at 4°C. The P815 cells (2x10^5^) were then mixed with purified NK cells (1x10^5^) and incubated for 4hr at 37°C, 5% CO_2_. After 4h of coincubation, the cells were stained with fluorochrome-conjugated anti-CD3, CD56 and CD107a. CD3- CD56+ cells were evaluated for the percentage of cell-surface CD107a expression.

**NK cell direct killing assay**

Infected primary CD4+ T cells were stained with a viability dye (AquaVivid; ThermoFisher Scientific) and cell proliferation dye (eFluor670; eBioscience) and used as target cells. Autologous purified NK cells, stained with another cellular marker (cell proliferation dye eFluor450; eBioscience), were added at different NK: target ratios (1:4, 1:2, 1:1) in 96-well V-bottom plates (Corning, Corning, NY). NK cells were preincubated in the presence of anti-NTB-A and anti-DNAM-1 or their matched IgG isotype control (10 μg/mL) prior being incubated with target cells. The plates were subsequently centrifuged for 1 min at 300 × g, and incubated at 37°C, 5% CO2 for 5 to 6 h before being fixed in a 2% PBS-formaldehyde solution. Infected cells were identified by intracellular staining for HIV-1 p24 as described above. Samples were acquired on an LSRII cytometer (BD Biosciences) and data analysis was performed using FlowJo vX.0.7 (Tree Star). The percentage of direct killing was calculated with the following formula: (% of p24+ cells in Targets) − (% of p24+ cells in Targets plus Effectors) / (% of p24+ cells in Targets) by gating on infected lived target cells.

**FACS-based ADCC assay**

Measurement of ADCC using a FACS-based assay was performed at 48h post-infection as previously described (1). Briefly, infected primary CD4+ T cells were stained with viability dye (AquaVivid; ThermoFisher Scientific) and cell proliferation dye (eFluor670; eBioscience) and used as target cells. Autologous PBMC effectors cells, stained with another cellular marker (cell proliferation dye eFluor450; eBioscience), were added at an effector: target ratio of 10:1 in 96-well V-bottom plates (Corning, Corning, NY). ADCC-mediating mAb 3BNC117 (0,5 µg/ml) was added to appropriate wells and cells were incubated for 15 min at room temperature. The plates were subsequently centrifuged for 1 min at 300 × g, and incubated at 37°C, 5% CO2 for 5 to 6 h before being fixed in a 2% PBS-formaldehyde solution. Infected cells were identified by intracellular staining for HIV-1 p24 as described above. Alternatively, effector cells were preincubated in the presence of anti-NTB-A and anti-DNAM-1 antibodies or their matched IgG isotype control (10 μg/mL) prior being incubated with target cells for blockade experiments. Samples were acquired on an LSRII cytometer (BD Biosciences) and data analysis was performed using FlowJo vX.0.7 (Tree Star). The percentage of ADCC was calculated with the following formula: (% of p24+ cells in Targets plus Effectors) − (% of p24+ cells in Targets plus Effectors plus Abs) / (% of p24+ cells in Targets) by gating on infected lived target cells.

**REFERENCES**

1. Richard J, Veillette M, Brassard N, Iyer SS, Roger M, Martin L, Pazgier M, Schon A, Freire E, Routy JP, Smith AB, 3rd, Park J, Jones DM, Courter JR, Melillo BN, Kaufmann DE, Hahn BH, Permar SR, Haynes BF, Madani N, Sodroski JG, Finzi A. 2015. CD4 mimetics sensitize HIV-1-infected cells to ADCC. Proc Natl Acad Sci U S A 112:E2687-94.

**Supplemental Figure legend**

**Fig S1. Experimental procedures.** CD4+ T lymphocytes were purified from resting PBMCs by negative selection using immunomagnetic beads. Purified CD4+ T cells were activated with phytohemagglutinin-L (PHA-L) (10 μg/ mL) for 48 hours and then maintained in RPMI 1640 complete medium supplemented with rIL-2 (100 U/mL) for 72 hours. Activated CD4+ T cells were then infected with VSV-G pseudotyped HIV-1 primary viruses by spin-infection. Twenty hours post-infection, infected cells were electroporated with selected siRNAs prior treatment with type I IFNs. Forthy-eight hours post-infection, the susceptibility of infected cells to NK cells responses, as well as cell-surface levels of BST-2, NTB-A and PVR were assessed by flow cytometry.

**Fig S2. Role of Vpu and Nef in HIV-1-mediated downregulation of cell-surface BST-2, NTB-A and PVR.** Primary CD4+ T cells were infected with CH58 T/F WT or variants defective for Nef and/or Vpu expression. Forty-eight hours post-infection, cells were stained with anti-BST-2, anti-NTBA or anti-PVR Abs, followed with appropriate secondary Abs. The graphs shown represent the median fluorescence intensities (MFI) detected on p24- cells for mock-infected cell and the infected population (p24+) for HIV-1-infected cells. Statistical significance was tested using unpaired t test (* p<0.05, ** p<0.01, *** p<0.001, **** p<0.0001, ns: non-significant).

**Fig S3. Effect of IFNβ on cell-surface CD4 levels.** Primary CD4+ T cells were infected with CH58 T/F, either WT or defective for Vpu or Nef expression. Twenty-four hours post-infection, infected cells were either mock-treated or treated for 24h with IFN-β. Forty-eight hours post-infection, cells were stained with anti-CD4 Abs, followed with appropriate secondary Abs. The graphs shown represent median fluorescence intensities detected on the p24+ population. Error bars indicate means +/- SEM. Statistical significance was tested using unpaired t test or Mann-Whitney test based on parametric test (ns: non-significant).

**Fig S4. Recognition of HIV-1-infected cells by bNAb 3BNC117 upon treatment with IFNβ.** Primary CD4+ T cells were infected with CH58 T/F WT (treated or not with IFN-β for 24h) or a variant defective for Vpu expression. Forty-eight hours post-infection, cells were stained with anti-Env bNAb 3BNC117 (0,5 μg/mL), followed with appropriate secondary Abs. Statistical significance was tested using unpaired t test (* p<0.05)
